# A novel, non-invasive cnidarian venom extraction device

**DOI:** 10.1016/j.toxcx.2025.100240

**Published:** 2026-01-16

**Authors:** Phillip J. Robinson, Steven A. Trim, Carol M. Trim

**Affiliations:** a The Deep, Tower Street, Hull, East Riding of Yorkshire, HU1 4DP, United Kingdom; b Natural Sciences, School of Science, Psychology, Arts and Humanities, Computing, Engineering and Sport, Canterbury Christ Church University, Kent, CT1 1QU, United Kingdom; c Venomtech Ltd, Building 500, Discovery Park, Sandwich, Kent, CT13 9NJ, United Kingdom

**Keywords:** Cnidaria, Jellyfish, Coral, Sea anemone, Venom, PLA2

## Abstract

Cnidaria represent one of the most ancient venomous lineages with thousands of extant species and their toxins have long been known to signify a source of therapeutic potential. Despite this recognition, cnidarian toxin research has progressed relatively slowly when compared to other taxa. One of the major factors for this slow development pertains to the difficulties involved with obtaining samples, particularly from benthic species which are sessile, where dissected tissues have historically been required. Additionally, the instability of marine venoms has further hindered progression of cnidarian venom research. The research presented aimed to address these issues through the design and development of a novel, non-invasive, venom extraction device that works on a range of cnidarian species. The device functioned underwater at depths ranging from 50 mm down to 5 m whilst scuba diving and was able to successfully obtain venom samples from all 12 species tested. These species were from three taxonomic groups Actiniaria (sea anemones), Scleractinia (corals) and Scyphozoan (Jellyfish) with four species from each. These venom samples revealed the expected phospholipase A2 activity but also the four Scleractinia venoms demonstrated phospholipase A2 inhibitory properties. This is the first description of phospholipase A2 inhibitory activity in cnidarian venoms and further work is required for full characterisation.

## Highlights

• An inexpensive, non-invasive device for collecting Cnidarian venom.
• Post harvest concentration of Cnidarian venom from seawater with cold acetone precipitation preserved venom activity.
• Phospholipase A2 activity from Cnidarian venoms collected and first indication of PLA2 inhibitory activity in Cnidaria.

## 1 Introduction

A multitude of approaches have been developed to obtain venom from the tissues of Cnidaria through the years. Initially dissected tentacles of *Chironex fleckeri* were placed onto human amnion in which an electrical current was passed across to elicit stimulation of nematocysts (Barnes, 1966; Senčič and Maček, 1990; Carrette and Seymour, 2004; Jouiaei et al., 2015; Jouiaei et al., 2025). The potential occurrence of cellular contamination from amniotic proteins within the sample generated a requirement to formulate new methods of extracting venom from Cnidaria. Many previous methods have potential issues such as, denaturation of proteins related to the extraction method, cell debris contamination of the samples, or sample filtration requiring laborious methodologies (Carrette and Seymour, 2004; Berking and Herrmann, 2006; Helmholz et al., 2007; Yanagihara and Shohet, 2012; Jouiaei et al., 2015; Jouiaei et al., 2025; Ponce et al., 2015; Cantoni et al., 2020). One of the quickest methods which has yielded uncontaminated samples was reported by Jouiaei et al. (2015) which employed the chemical discharge of nematocysts using ethanol. This extraction method paved the way for the development of this novel extraction device which utilises ethanol to elicit nematocyst discharge *in situ* whilst proving to be non-invasive to the species concerned. The authors previously determined the exposure time required for ethanol to fully stimulate nematocyst firing and protein release using autotomised tentacles (Robinson et al., 2019). In that study nematocyst firing ceased after 20 s and protein concentration rose linearly over 8 min. The protein concentration rate is assumed to be predominantly though diffusion. Therefore, the collection device needs to be in contact with the nematocysts for at least 20 s and include a flow element to reduce the lag from diffusion.

Alongside the development of a new extraction method, the storage and preparation of extracts was also considered, as cnidarian toxins are somewhat unstable and tend to start degrading soon after being extracted (Brinkman and Burnell, 2009; Nagai et al., 2002; Ponce et al., 2016; Xie et al., 2017). Previous studies have used: ammonium sulphate; trifluoracetic acid; trichloroacetic acid; desalting columns; and gel filtration chromatography to desalt the venom extracts and preserve the activity (Monastyrnaya et al., 2002; Martins et al., 2009; Gallagher, 2012; Cassoli et al., 2013; Lazcano-Pérez et al., 2017; Rastogi et al., 2017; Orts et al., 2018; De Domenico et al., 2019). Degradation is even reported to occur during storage in temperatures as low as −37 °C, with current literature showing a marked decline in the activity of protease and phospholipase A2 (PLA2) in a timespan of one and three weeks, respectively (Gusmani et al., 1997; Šuput, 2009; Rastogi et al., 2017). It would be reasonable to assume that other enzymatic activity may also be at risk of degradation and therefore highlight a need to process samples and undertake assays in a time dependant manner. This study examined spin columns and cold acetone precipitation as rapid methods of desalting and preserving venom activity.

PLA2 enzymes have been recruited into a diverse range of venom systems from well-known Hymenoptera, though to snakes, scorpions and spiders (Trim et al., 2021). Within Cnidaria less is known due to the sampling limitation highlighted. PLA2 activity within Actiniaria has been reported with 69 individual PLA2 sequences deposited in UniProt (Ahmad et al., 2025). Specific examples include those from *Actinia equina* (Alcaide et al., 2024) and *Entacmaea quadricolor* (Hoepner et al., 2024). PLA2 from Scleractinia coral is the most abundant in UniProt with 111 entries (Ahmad et al., 2025), however, many of these come from Pocillopora and this study used tissue lysates (Nevalainen et al., 2004). Within the Scyphozoan species PLA2 activity is known from Aurelia and Cassiopea (Radwan et al., 2001), there are only five PLA2 proteins deposited in UniProt (Ahmad et al., 2025) from Scyphozoa and only one of those is reviewed (Lotan et al., 1995). The other four sequences are marked for consideration of removal in 2026 as they remain unreviewed, two from Rhopilema esculentum (accession numbers A0A0H3W5L1 & X2G7F0) that have an incomplete reference cited and two from Nemopilema nomurai (accession numbers A0A1D8GZE6 & A0A1D8GZE4) Cited as Heo et al. (2016). However, PLA2 immunoreactivity has been found in many Scyphozoa (Lotan et al., 1996).

## 2 Material and methods

### 2.1 Ethical considerations

Despite the exclusion of non-Cephalopod invertebrates under the Animals (Scientific Procedures) Act 1986, all work undertaken during this research project was subject to an ethical review at The Deep Aquarium prior to commencement and then performed in a way that ensured minimal stress and damage was caused to the research subjects. The non-invasive nature of ethanol extractions is supported by research carried out by Pascoe et al. (2003) who concluded that full emersion in solutions of ethanol with a concentration of up to 10 mg/L generated a minimal retraction in polyps and elicited only a slight reduction in the polyp response. The time in which the polyps were in contact with ethanol in the Pascoe study disproportionately surpasses the duration in which the cnidarians were in contact with ethanol during the extractions presented here.

### 2.2 Design & manufacture of a novel venom extraction device (nVED)

**Fig. 1:**
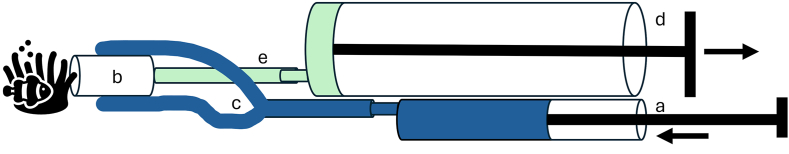
The schematic for the novel extraction device (nVED), highlighting all major components and fluid flow. The 20 ml syringe (a) is pre-filled with firing solution (blue) 100 % ethanol or isopropanol before submerging. Once submerged the air is purged from the firing chamber (b) and this is then placed over the target tissue. As the plunger is depressed on the 20 mL syringe (a) an alcohol chemocline is established in the firing chamber through the flow of firing solution through the junction (c). This stimulates firing of the nematocysts and the release of venom (green) which is collected into the 60 mL sample chamber (d) through line e. (For interpretation of the references to color in this figure legend, the reader is referred to the Web version of this article.)

**Fig. 2:**
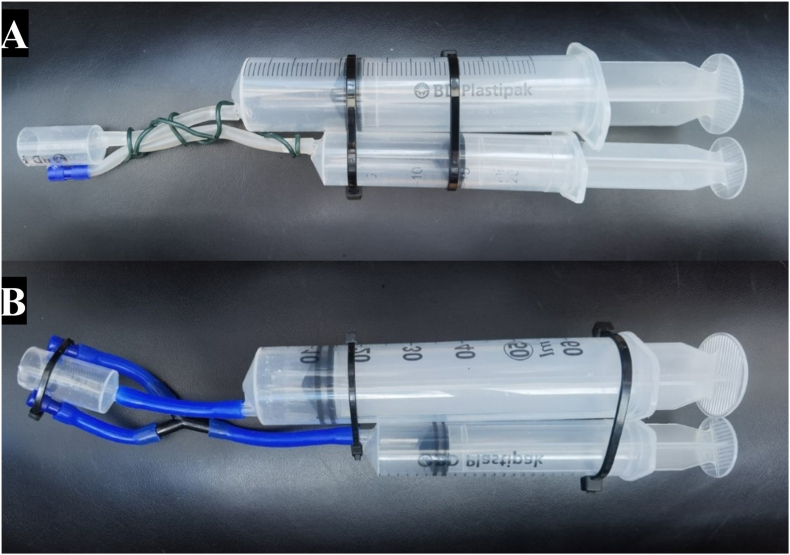
Photographs of the nVED devices used in this study. [A] The prototype device used for initial timed extractions and protocol testing. [B] version 2 of the device with two firing solution delivery ports and improved connections. 13 individual nVED devices of version 2 were manufactured: one being dedicated to each species to prevent contamination.

The nVED was designed in three main parts (Fig. 1), which were interlinked using 6 mm silicone aquarium airline (Marina, Hagen, Castleford, UK), and a 90° elbow, with a further refinement also consisting of a 6 mm Y-connector and second 90° elbow (Algarde Aquatic Products, Norwood Aquarium Ltd, Surrey, UK). The main body, and working parts, of the nVED was constructed using three syringes (BD Plastipak, BD UK 306 Ltd, Berkshire, UK): a ‘firing chamber’, a ‘sample chamber’ and a ‘collection chamber’. The firing chamber, held a ‘firing solution of either 100 % ethanol or 100 % isopropanol (IPA), in a 20 mL syringe. Ethanol under licence from Venomtech Ltd was used for the first proof of concept extractions, and 100 % isopropanol was used for the full study as it was cheaper and doesn't require a licence for use in the UK. The sample chamber consisted of a 60 ml syringe designed to collect the extracted venom along with the firing solution and some sea water. The final piece was the collection chamber, which consisted of a 10 ml syringe that had the plunger removed and was cut in half. This chamber provided the enclosed area in which the tissues are exposed to the firing solution, releasing the venom. To build the nVED, the firing and sample chambers were cable tied together to form the main body of the device and two lengths of 6 mm airline measuring ∼100 mm were attached to the tip of each syringe. A 6 mm hole was drilled into the wall of the collection chamber ∼8 mm from the bottom edge of the chamber, to which one end of the 90° elbow was inserted. The tip of the collection chamber was then attached to the opposite end of the airline which had been connected to the sample chamber and the 90° elbow was attached to the opposite end of the airline which had been attached to the firing chamber. In the first version of the device (Fig. 2a), a length of gardening wire was wrapped around the airline to offer some stability when sampling. This was not repeated in the second iteration (nVED2) (Fig. 2b), which had a Y-connector installed along the length of the airline that was attached to the firing chamber. The two ends of the Y-connector were then attached to the 90° elbows in the collection chamber, which had a second 6 mm hole drilled on the opposite side of the collection chamber with another 90° elbow inserted (also see supplementary videos on construction and use of the nVED).

### 2.3 Underwater venom extraction

**Fig. 3:**
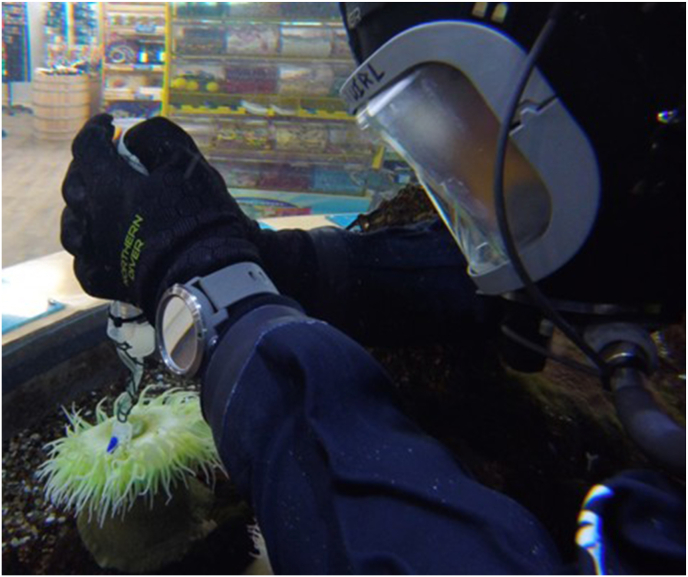
The prototype extraction device being used to sample *A. xanthogrammica* during the in the first trial for suitability of use in a dive situation.

Prior to transporting the extraction nVED to the area of sampling, the firing chamber was loaded with the firing solution. In line with general dive safety procedures, assessments of the conditions were carried out, evaluating the flow rates and animal behaviours within the system if collecting *ex situ* in aquaria. When approaching *in situ* extractions the tidal activity and surroundings must be assessed. Once submerged, the nVED was inverted to purge the airlock from the collection chamber. The collection chamber of the nVED was positioned away from the flow of the water to minimise the loss of firing solution when extraction was undertaken (Fig. 3). The collection chamber was placed over the target tissue to be extracted from, these were prey capture tentacles for Actiniaria and Scyphozoa with small a polyp containing branch used for Scleractinian corals. The plunger of the firing chamber was slowly depressed, observing the formation of a chemocline within the collection chamber whilst ensuring that there was no loss of firing solution from the collection chamber. Once a chemocline had been observed within the collection chamber, the plunger of the sample syringe was slowly drawn back whilst simultaneously continuing to depress the plunger of the firing chamber. It was important to keep a balance between the drawing of the plunger of the sample chamber and expelling the firing solution out of the firing chamber, ensuring that the process was slow enough to maintain a constant visible chemocline within the collection chamber. The average time of each draw was planned to last around 60–90 s, longer than the time required to fully discharge the nematocysts compared to autotomised tentacles (Robinson et al., 2019) and collect the venom solution. Once the firing chamber was depleted, there was a brief continuation of the draw into the sample syringe until the chemocline within the collection chamber had disappeared. Once the extraction was complete, the nVED was removed from the water and the contents of the sample syringe were expelled into a 50 mL centrifuge tube and immediately stored at −20 °C.

### 2.4 Species used

Venom extracts from 12 different species were separated into three groups of four species. Four samples coming from Actiniaria, four samples from Scleractinia and four Scyphozoan species. The species of actiniarian sea anemones were *Actinia equina*, *Anthopleura xanthogrammica*, *Entacmaea quadricolor*; and *Thalassianthus aster*. The Scleractinian coral species used were *Catalaphyllia jardinei*, *Euphyllia glabrescens*, *Fimbriaphyllia ancora*, and *Seriatopora caliendrum*. The Scyphozoan jellyfish species were *Aurelia aurita*, *Cassiopea xamachana*, *Chrysaora colorata*, and *Chrysaora pacifica*. All animals involved in this study are all long-term captive specimens that have been extensively cultured and shared between multiple high level and reputable aquaria within the UK rather than sourced specifically for this study. Identification was confirmed by the authors and multiple experienced Aquarists, previously.

### 2.5 Venom purification for storage & transportation

Two methods of desalting and protein concentration were tested. Smaller samples were purified using 0.5 mL 10,000 Da molecular weight cut-off (MWCO) centrifugal filters (Amicon® Ultrafree®-MC). Each 10 kDa filter was centrifuged (Hermle Z160M) for 10 min at 5000×*g*. The concentrates, which were retained within filter tubes after each spin, were then pooled into separate 0.5 mL centrifuge tubes for each species.

For larger samples, 10 mL of fresh extract was aliquoted into a 50 mL tube containing 40 mL of cold (−20 °C) acetone (Simpson and Beynon, 2010) and agitated vigorously before being transferred back into storage at −20 °C overnight or until required. Precipitate was collected via filtration through 11 μm qualitative filter paper (Whatman PLC, Cytiva, Global Life Sciences Solutions Operations UK Ltd, Sheffield, UK) and the precipitate transferred into 1.5 mL centrifuge tubes. Acetone precipitation was determined to be the most effective as the other methods failed to yield enough protein for characterisation.

Following either method of purification, the samples were lyophilised, in an airtight 530 mL food-grade container (Sistema® To Go™, Sistema® Plastics UK Limited, Surrey, UK) that was half filled with loose silica gel (SiO_2_) (Disidry, The Aerodyne s. n.c, Italy). The lid of the container was wrapped with Parafilm® to guarantee that the container remained as airtight. Samples were desiccated at an ambient temperature of ∼26 °C until they had been fully lyophilised. Once lyophilisation had been achieved, samples were then stored at −20 °C.

### 2.6 Venom characterisation

Total protein was determined through spectrophotometry at 280 nm using the DS-11 microspectrophotometer (DeNovix, Wilmington, USA). The PLA2 activity of the processed venom extracts were assessed using an EnzChek® PLA2 assay kit (Invitrogen Ltd, Paisley, UK) as per the manufacturer instructions with volumes reduced to 20 μL final volume for use in 384 well plates. These were read in a FLUOstar Galaxy plate reader (BMG Labtech Ltd, Buckinghamshire, UK) with an excitation of 485 nm and emission of 520 nm. Cnidarian venoms were diluted to 100 ng/μL and 750 ng of each venom was used in triplicate for the activity and inhibition experiments. The positive control was Honeybee (*Apis mellifera*) venom which was provided in the kit and diluted to 5 U/mL. The reaction volume consisted of 12.5 μL PLA2 substrate solution and 7.5 μL reaction buffer or venom to each well of a 384 well black plate (781076, Greiner bio-one) with the positive control, negative control (tank water), or sample. The assay was also performed in inhibitor mode where the venom was pre-incubated for 10 min at 25 °C with the bee (*Apis melifera*) venom positive control before the substrate was added. Data was analysed by first subtracting the baseline levels detected in the system water for each species and comparing to the standard 5 U/ml PLA2 in the standard *Apis melifera* venom for PLA2 activity. PLA2 inhibition was calculated as percentage activity verses uninhibited *Apis melifera* venom standard. Statistical relationships were assessed using a two-sided, equal variance students’ T test in Excel (Microsoft).

## 3 Results

### 3.1 *In situ* venom extractions

The initial work was carried out through reaching into the specimen aquaria with the device, later work proved it to be practical in a diving scenario (Fig. 3). The collected samples were confirmed as non-invasive through light microscopy, no nematocysts or cellular material were detected, only small amount of mucous and salt crystals were present.

**Fig. 4:**
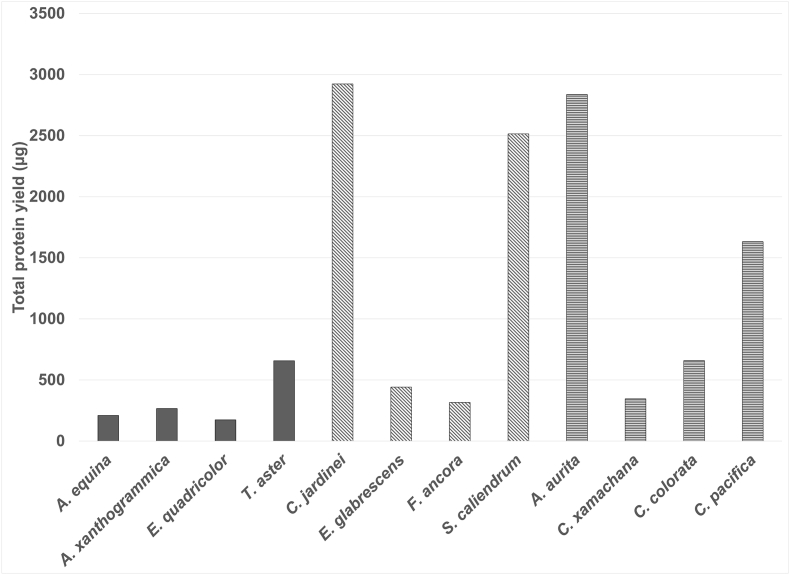
The protein yield obtained from the venom extract of each species after acetone precipitation. The Actiniarian Species (solid bars) produced the lowest yields, with the lowest yield (174 μg) coming from *Entacmaea quadricolor*. The Scleractinia (diagonal stripes) performed better with over 2000 μg from two species. 50 % of The Scyphozoan species (horizontal stripes) were high yielding, however the *Cassiopea xamachana* were low yielding at 345 μg.

Variation in average extraction duration and yield from each taxonomic group is shown in Table 1. The average extraction duration across all samples (n = 19) was 76 ± 47.07 s. There were a number of variables that affected the extraction time for example, position of the animal, motility of the animal, the flow in the system, shape and size of the extraction site, depth in which the animal is located, and access space to undertake the sampling. With a duration of 46 s, the extraction undertaken on *A. xanthogrammica* when diving was the quickest and most straightforward extraction to perform, whilst other actiniarian samples were undertaken with relatively few complications beyond their ability to retract tissues rapidly, resulting in the occasional need to reposition the collection chamber over fresh tissue of the same animal. Conversely, the sampling of Scyphozoa remained problematic throughout, with a maximum time of 255 s taken for a single *A. aurita* extraction. Scleractinian samples proved to be free of any complications, with tissues easily accessible and no real need to reposition the collection chamber throughout the extraction. Total protein yields for each species, following purification through acetone precipitation, varied by species but there was no significant difference between the taxonomic groups (Fig. 4). The actiniarian species produced the lowest yields (Table 1), All species yielded over 100 μg per extraction (Fig. 4) and therefore a suitable amount of venom protein was collected for subsequent analysis. Average yield was 1081 μg however the median (549 μg) reflects the variability and only 30 % of species tested yielded more than a milligram of total protein. With the information currently available it is not clear whether the source of the inter-species variability is due to the intrinsic biology of the organisms, the ability of the nVED to suitably enclose the nematocyst bearing tissue as destructive sampling was not possible in this study or some other factor.

**Table 1:** Average extraction duration and yield for each taxonomic group.

Group	Average Extraction duration (s)	Average Extraction yield (μg)	n
Actiniara	77 ± 19.34	327 ± 223	8
Schyphozoa	97 ± 80.98	1368 ± 1121	5
Scleractinia	62 ± 21.57	1548 ± 1361	6

**Fig. 5:**
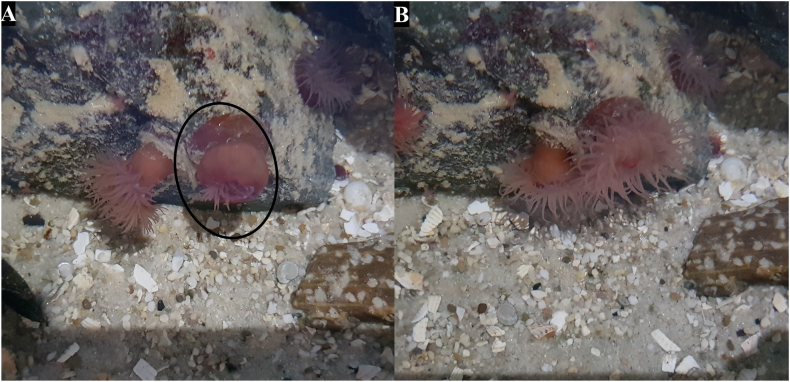
Post-extraction (PE) recovery of *A. equina*. [A] 1-min PE, the entire anemone (circled) has retracted in a similar way to a normal response to touch or prey capture. [B] 6 h PE, a full recovery has been made within this duration and as such, no follow up images were necessary.

**Fig. 6:**
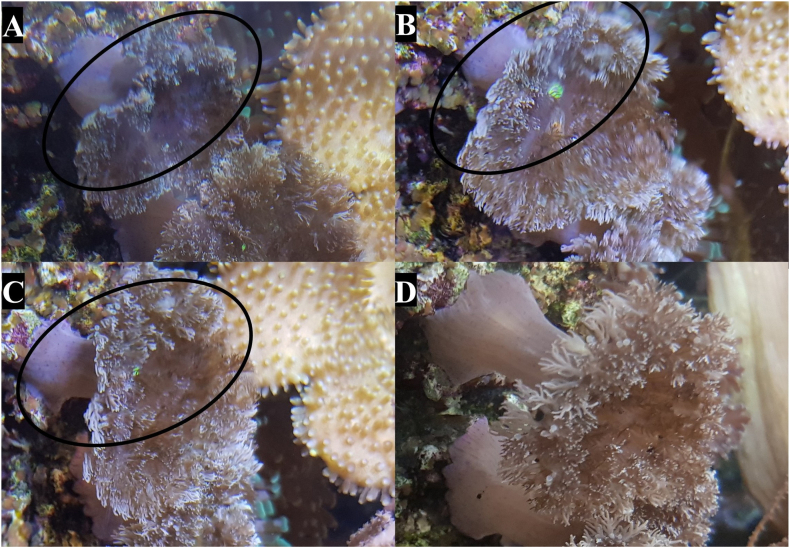
Post-extraction (PE) recovery of *T. aster*. [A] 1-min PE, visible reduction in the left side of disc where extraction took place (circled). [B] 2 h PE, some recovery of disc size but still a noticeable reduction from the unsampled areas. [C] 22 h PE, disc size has made a return to pre-extraction size but there is some observable reduction in dendritic tentacles. [D] 44 h PE, disc and tentacles have made a full recovery and returned to pre-extraction sizes.

**Fig. 7:**
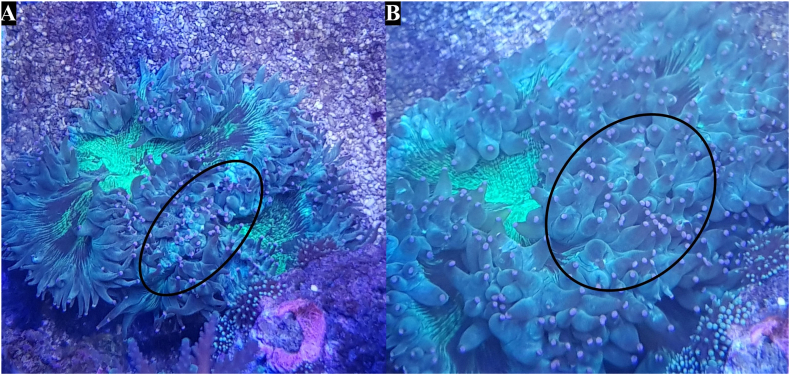
Post-extraction (PE) recovery of *C. jardinei*. [A] 1-min PE, an observable retraction of tentacles occurs in the sampled area (circled), however this is superficially consistent with the retraction observed through general contact. [B] 6 h PE, complete recovery of sampled area, with all tentacles returning to a pre-extraction state.

**Fig. 8:**
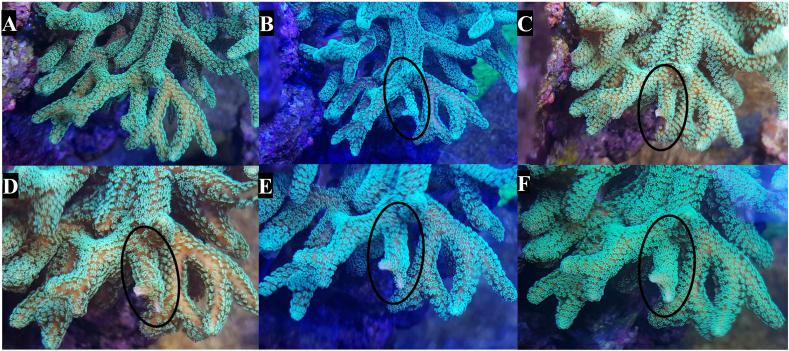
Post-extraction (PE) recovery of *S. caliendrum*. [A] The pre-extraction image shows the small polyps and calcified skeleton which make it difficult to observe significant changes. [B] 10 min PE, the entire branch (circled) exhibits some polyp retraction. [C] 28 h PE, the polyps at proximal end of the branch are recovering but the distal end still shows a marked retraction of polyps. [D] 50 h PE, much of the branch has made a recovery but the polyps around the two tips are still retracted. [E] 72 h PE, the tip on the right side of the branch shows some polyp extension, although still decreased. [F] 170 h PE, both sides of the tip now exhibit polyp extension which, although diminished compared to the pre-extracted state, have been accepted as a recovery, given the duration of time and the lack of any tissue necrosis.

Post-extraction (PE) follow-up was performed on the individual species used where practical. Complete recovery was deemed as visual return of phenotype to pre-extracting morphology of tentacle or polyp extension. Of the actiniarian species, *A. equina* made a full recovery within 6 h with no noticeable difference between extracted tentacles and control (Fig. 5) and *T. aster* had made a full recovery after 2 days (Fig. 6). No visible change was detected in *A. xanthogrammica* (data not shown). Scleractinia species recovered well from the extraction with no tissue injury detected although recovery time was very different. *C. jardinei* which had made a full recovery after 6 h (Fig. 7). Post-extraction polyp retraction in *S. caliendrum* was prolonged with decreased extension over 72 h (Fig. 8). No visible change was detected in *F. ancora* (data not shown). The pelagic nature of Scyphozoan species hampers individual identification and thus individual post-extraction follow-up was not achieved. There were no issues noticed in the tanks post extraction by the authors or the aquarists performing their standard checks. The minor physical changes caused by the extractions were generally short lived, with most returning to a visual normal in under three days with no long-term effects observed. These results further correlate with multiple sources which emphasize the considerable regenerative capability of cnidarians (Pascoe et al., 2003; Porat and Chadwick-Furman, 2005).

### 3.2 Venom activity analysis

**Fig. 9:**
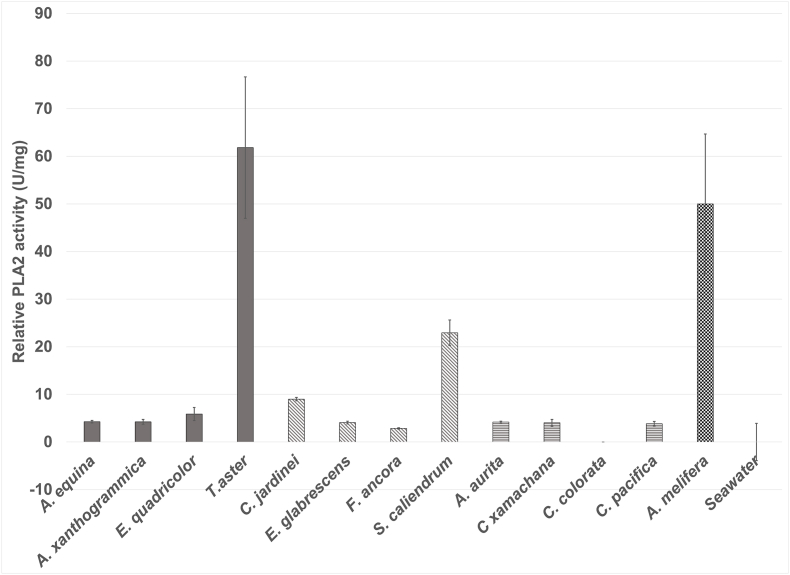
Relative PLA2 activity detected from aquariumcollected venoms, were significantly higher than the background artificial seawater for the majority of species. Actiniaria species (solid colour) *A. equina* p = 0.0444, *A. xanthogrammica* = 0.0453, *E. quadricolor* p = 0.02400, *T. aster* 0.0404, Scleractinia species (diagonal stripes) *C. jardinei* p = 0.0231, *E. glabrescens* n. s. p = 0.0514.*F. ancora n. s.* p = 0.13, *S. caliendrum* p = 5.76 × 10^−5^. Scyphozoan species (horizontal stripes) *A. aurita* 0.04777. *C. xamachana* n. s. p = 0.052, *C. colorata* n. s. p = 0.7, *C. pacifica* n. s. p = 0.06. Controls displayed in checked bars, positive control of 5 U/ml *Apis melifera* venom and negative control of artificial sea water.

**Fig. 10:**
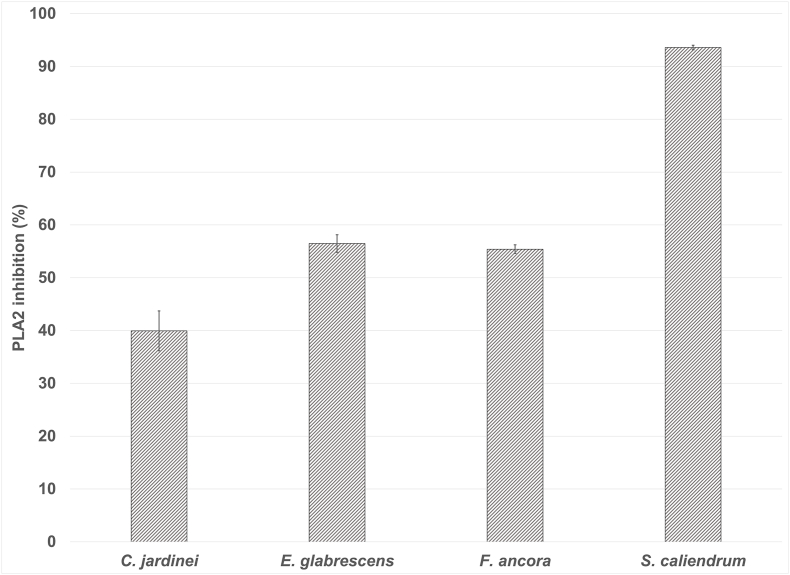
Significant inhibition of bee venom PLA2 was detected in the four Scleractinian species - P values for PLA2 inhibition -*C.jardinei* p = 0.0454, *E. glabrescens* = 0.000744, *F. ancora* = 0.00095755. and *S. caliendrum* p = 5.2092 × 10^−5^.

Detectable levels of PLA2 activity were observed (Fig. 9) although these were not statistically significant in five of the species. All of the actiniarian species had significant (p-value =< 0.05) detectable PLA2 activity with the highest of 61.8 U/mg protein detected in *T. aster*. The Scleractinian, *Seriatopora caliendrum* also had high PLA2 activity of 22.9 U/mg protein. It was hypothesised that the low detectability of PLA2 activity could also be due to PLA2 inhibitors being present in the venom. Significant PLA2 inhibition activity was detected in three of the four Scleractinian species (Fig. 10) but this activity was not detected in the other two groups tested (data not shown). 750 ng of these cnidarian venoms were able to inhibit 37.5 microunits of *Apis melifera* PLA2 activity from a 10 min pre-incubation. *S. caliendrum* is the only species where both PLA2 activity and inhibition were detected in the same sample. Further work to fractionate these venoms would be required to see the full extent of PLA2 activity and identify potential inhibitors, as well as other activities.

## 4 Discussion

Obtaining cnidarian venom samples has historically been problematic in many respects and there was a clear need for a means to bridge this gap. The discovery of chemical extraction techniques, which lead to a progression in the ability to carry out rapid venom extractions in Cnidaria, created a niche for the design of a device which could utilise this method of extraction *ex situ* in aquaria and potentially *in situ*. The results of this research suggest that there is great potential for a novel extraction device to forward the current knowledge of cnidarian toxins through expanding the number of species which may be sampled. The outcome provides a means of continuation from the innovative work on chemical extraction of venom from *Chironex fleckeri*, undertaken by Jouiaei et al. (2015) , through the combination of ethanol extractions with the nVED which is easy and cheap to manufacture. Through this work the authors have demonstrated the nVED was effective in collecting protein from cnidarian tentacles and these proteins had activity expected from venom. Additionally, the need to desalt and purify samples is an often-essential step that is involved with undertaking assays of marine toxins, this is largely due to their instability and high mucous content (Razpotnik et al., 2010; Frazão et al., 2012; Xie et al., 2017). Establishing the cold acetone precipitation method of desalting and concentrating the venom extract was a key enabling factor in the research. Although comparison of other methods was outside the scope of this study, the10 kDa MWCO desalting columns probably failed due to loss of smaller peptides. It is expected that use of the nVED could benefit future Cnidaria toxin research from both a toxinological and a drug discovery standpoint. The current design, however, should be presented for some consideration as there are currently limitations when accounting for taxa-wide inclusion and there is a need to explore a range of variations in the future. Modifications and species-specific revisions to the device will be required in order to work with different species, for example, the sampling of large plating species of Scleractinia, such as *Montipora* spp., as the morphology of these corals would not currently allow the containment of tissues within the collection chamber. Sampling of Scyphozoan species did present challenges in which the process of securing and retaining tentacles in the collection chamber proved problematic. This would likely transfer to pelagic species that were outside the scope of this research such as, Cubozoa and Hydrozoa. These limitations would currently lead to reduced yields or an inability to obtain samples altogether, unless they were restrained. The authors anticipate that the size of the extraction chamber can be scaled to suit other target species. Other revisions could include, changing the number and position of the ethanol delivery ports, altering the shape of the extraction chamber, and using an eccentric syringe cut diagonally as an extraction chamber to maximise the contact area for plating coral species such as, *Montipora* spp.

Through the design and further development of the nVED it is hoped that there will be an advance in the current understanding of cnidarian toxins and, as a result of this, the discovery of a range of novel therapeutics which have remained untapped until this point. Furthermore, it is hoped that the potential discovery of drug leads as a result of this device may also further highlight the economic importance of Cnidaria, consequently providing an emphasis on the need for additional conservation measures to mitigate the loss of biodiversity and safeguard the source of future therapeutics (Braat and Groot, 2012; Lin et al., 2018; Cicka and Quave, 2019; Robinson et al., 2021).

Through the use of the nVED the authors have been able to confirm expected PLA2 activity in many of the cnidarian species sampled but have also detected PLA2 activity in species not previously reported, such as *Thalassianthus aster* and the *Seriatopora caliendrum*. No PLA2 inhibitors have been published for any of the species investigated in this study, such that the PLA2 inhibitory activity detected in the four Scleractinia species tested represents new findings for these species.

Although a comparison of methods to recover cnidarian venom, undertaken by Cantoni et al. (2020), may highlight downfalls in the chemical induced recovery methods previously seen with Jouiaei et al. (2015). Their conclusion that the methods reviewed were ineffective and impractical means of venom extraction may potentially lead to a significant array of missed opportunities for the future of cnidarian venom research. Despite the results found with current chemical recovery methods falling short of older techniques, there are several beneficial aspects associated with chemical extraction that are not necessarily obtained through more intrusive methods. The development of an effective chemical recovery using the nVED method allows for a substantial amount of sampling freedom due to the anticipated ability to perform *in situ* extractions on an array of species within a given location. Moreover, the non-invasive approach utilised by the device presented within this research, may increase the likelihood of successfully acquiring permits to bio-prospect species that have remained, up until this point, an untapped source of novel toxins and as this paper demonstrates it has the ability to be used within public aquaria without harming the specimens on display.

## 5 Conclusion

The nVED device designed within this research was successful in collecting venom samples in a non-invasive manner, yielding detectable levels of protein with venom like PLA2 activity and novel PLA2 inhibitory activity, many of which have not previously been reported. These results indicate that the nVED device design is functional and can be used to obtain viable samples from *in situ* organisms. When coupled with cold acetone precipitation these venoms can be stored and transported for later functional assays.

## CRediT authorship contribution statement

**Phillip J. Robinson:** Writing – original draft, Methodology, Investigation, Data curation. **Steven A. Trim:** Writing – review & editing, Supervision, Methodology, Formal analysis, Data curation, Conceptualization. **Carol M. Trim:** Writing – review & editing, Supervision, Methodology, Investigation, Formal analysis, Data curation.

## Ethical statement

Despite the exclusion of non-Cephalopod invertebrates under the Animals (Scientific Procedures) Act 1986, all work undertaken during this research project was subject to an ethical review at The Deep Aquarium prior to commencement and then performed in a way that ensured minimal stress and damage was caused to the research subjects. The non-invasive nature of ethanol extractions is supported by research carried out by Pascoe et al. (2003) who concluded that full emersion in solutions of ethanol with a concentration of up to 10 mg/L generated a minimal retraction in polyps and elicited only a slight reduction in the polyp response. The time in which the polyps were in contact with ethanol in the Pascoe study disproportionately surpasses the duration in which the cnidarians were in contact with ethanol during the extractions presented here.

## Declaration of competing interest

The authors declare that they have no known competing financial interests or personal relationships that could have appeared to influence the work reported in this paper.

## Data Availability

Data will be made available on request.
